# Supplementary material for: Obsidian forms by slow cooling
Source: Nat Commun. 2026 Feb 27;17:3266. doi: 10.1038/s41467-026-70110-1 (PMC13065812; doi:10.1038/s41467-026-70110-1)
Supplement: Supplementary file 1 — Supplementary Information [file 41467_2026_70110_MOESM1_ESM.pdf]

Supplementary Information

Composition of AOQ8 (from Allabar et al., 2022):

Supplementary Table 1. AOQ8 composition

Oxide	Wt.%
SiO_2	70.05
Al_2O_3	12.45
Na_2O	12.28
K_2O	5.23

Experimental data derived from analysis of tomography.

Supplementary Table 2. Vesicularity data

Time from onset of heating [seconds]	Vesicularity [%]
0	0.97
2905	1.36
3031	1.71
3118	2.38
3202	3.21
3244	3.81
3325	5.69
3410	7.5
3452	8.26
3537	9.61
3622	10.77
3664	11.29
3746	12.16
3830	12.94
3957	13.83
4043	14.35
4166	14.98
4334	15.57
4504	15.9
4670	16.28
4883	16.4
7949	13.87
8982	12.84
9151	12.72
9323	12.62
9405	11.87
9487	10.25
9572	8.65
9657	7.33
9741	6.25
9826	5.48
9908	4.98
10035	4.61
10246	4.49
10667	4.52
10833	4.51

The following plots are equivalent to Figure 4a-c in the main text, which we consider to be under canonical conditions. For each figure in the SI, we vary one of the canonical conditions – see *Supplementary Table* 3.

Supplementary Table 3. Conditions for model runs

Figure	p [MPa]	N_b [m^{-3}]	T_e [°C]	Varying
4a (main text)	1	10^{14}	825	Canonical
4b (main text)	1	10^{13}	825	
4c (main text)	1	10^{14}	825	
S1a	0.1	10^{14}	825	Pressure
S1b	0.1	10^{13}	825	
S1c	0.1	10^{14}	825	
S2a	10	10^{14}	825	
S2b	10	10^{13}	825	
S2c	10	10^{14}	825	
S3a	1	10^{14}	875	Emplacement temperature
S3b	1	10^{13}	875	
S3c	1	10^{14}	875	
S4a	1	10^{14}	950	
S4b	1	10^{13}	950	
S4c	1	10^{14}	950	
S5a	1	10^{12}	825	Bubble number density
S5b	1	10^{11}	825	
S5c	1	10^{12}	825	
S6a	1	10^{13}	825	
S6b	1	10^{12}	825	
S6c	1	10^{13}	825	

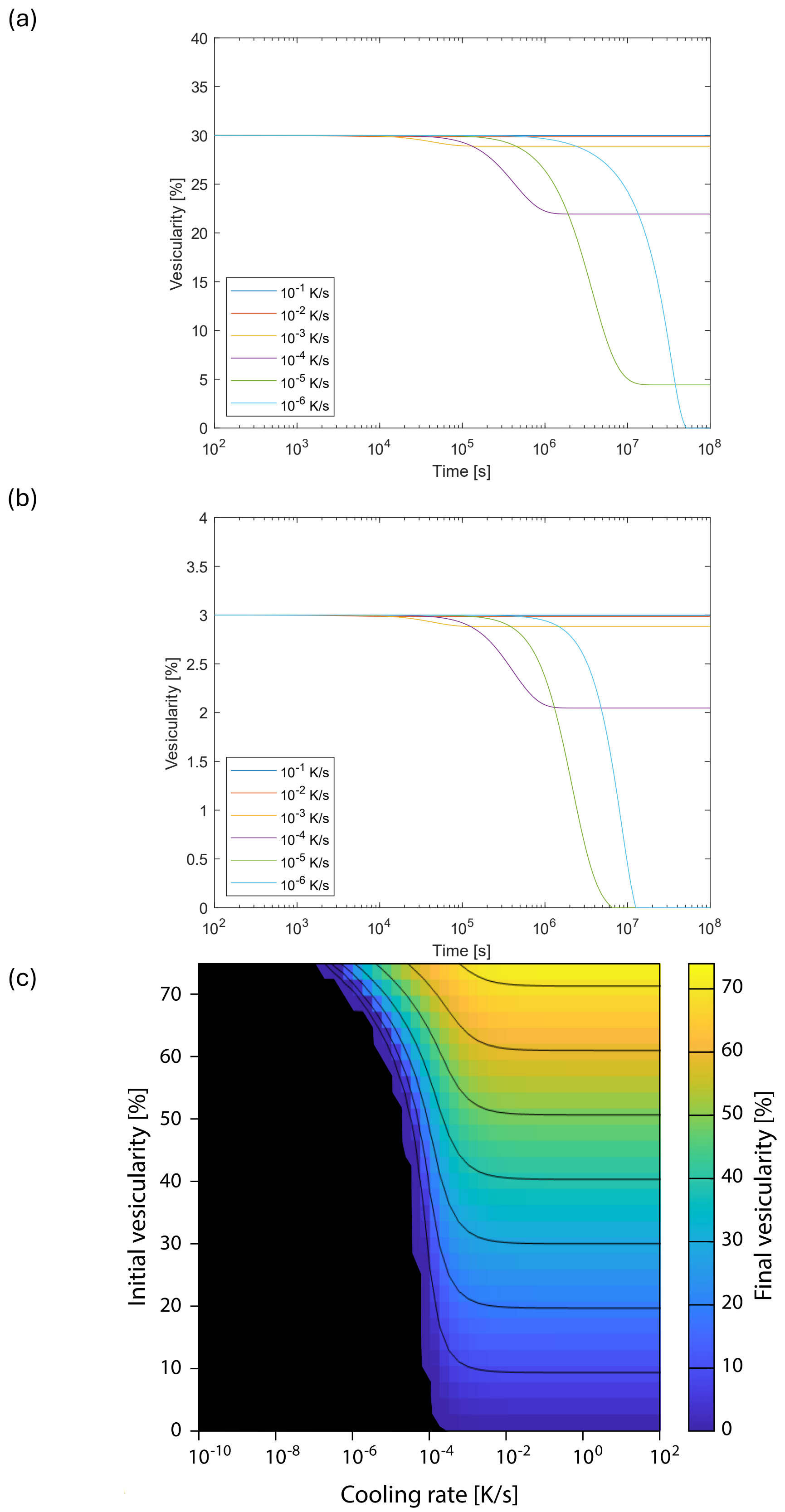

Supplementary Figure 1. Results of numerical modelling. *(a, b) Evolution of vesicularity over time for different cooling rates, for rhyolite samples starting from 30 and 3 vol. % vesicularity. (c) Map of initial and final vesicularity for rhyolite samples cooled at different rates. See Table S3 for conditions.*

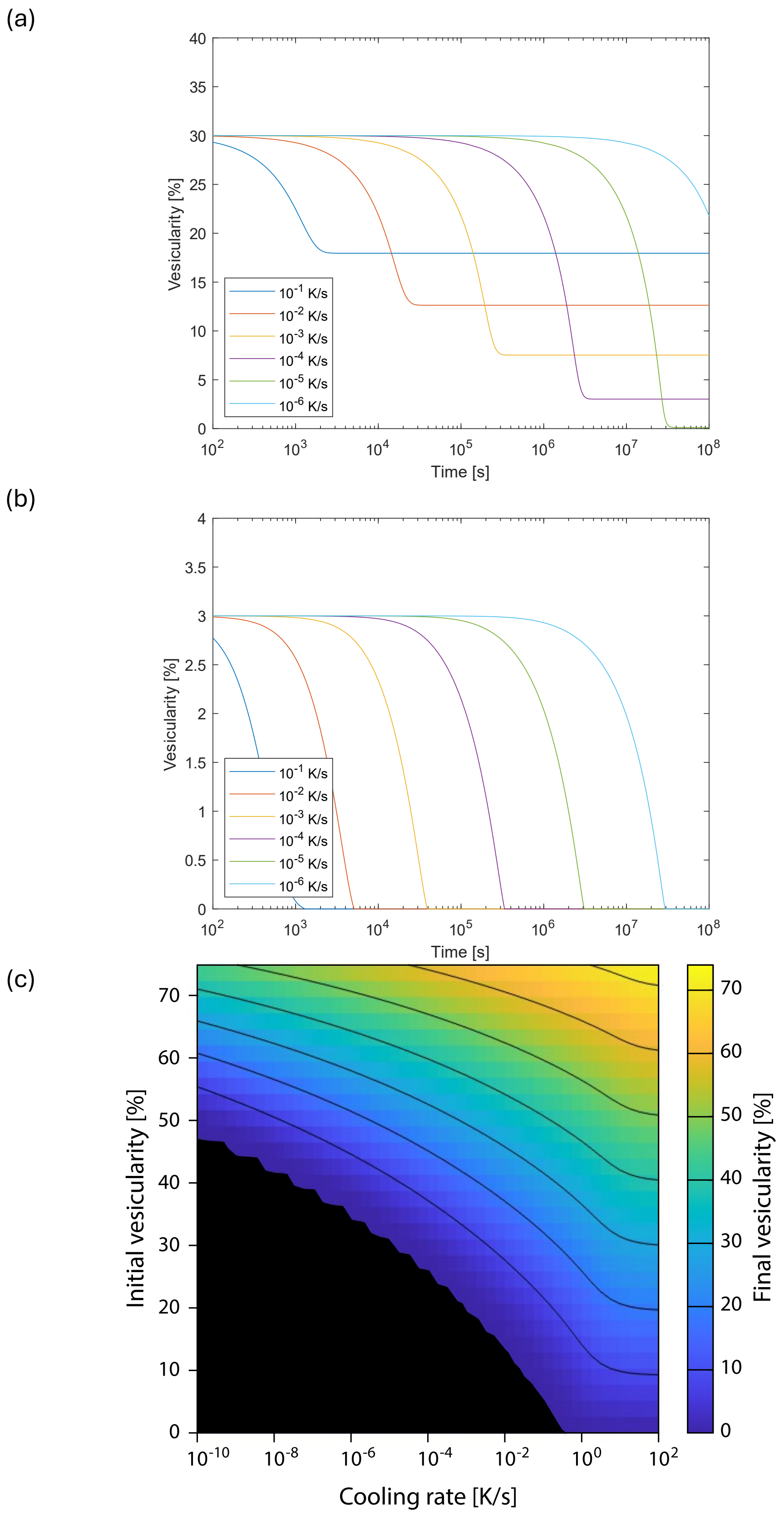

Supplementary Figure 2. Results of numerical modelling. *(a, b) Evolution of vesicularity over time for different cooling rates, for rhyolite samples starting from 30 and 3 vol. % vesicularity. (c) Map of initial and final vesicularity for rhyolite samples cooled at different rates. See Table S3 for conditions.*

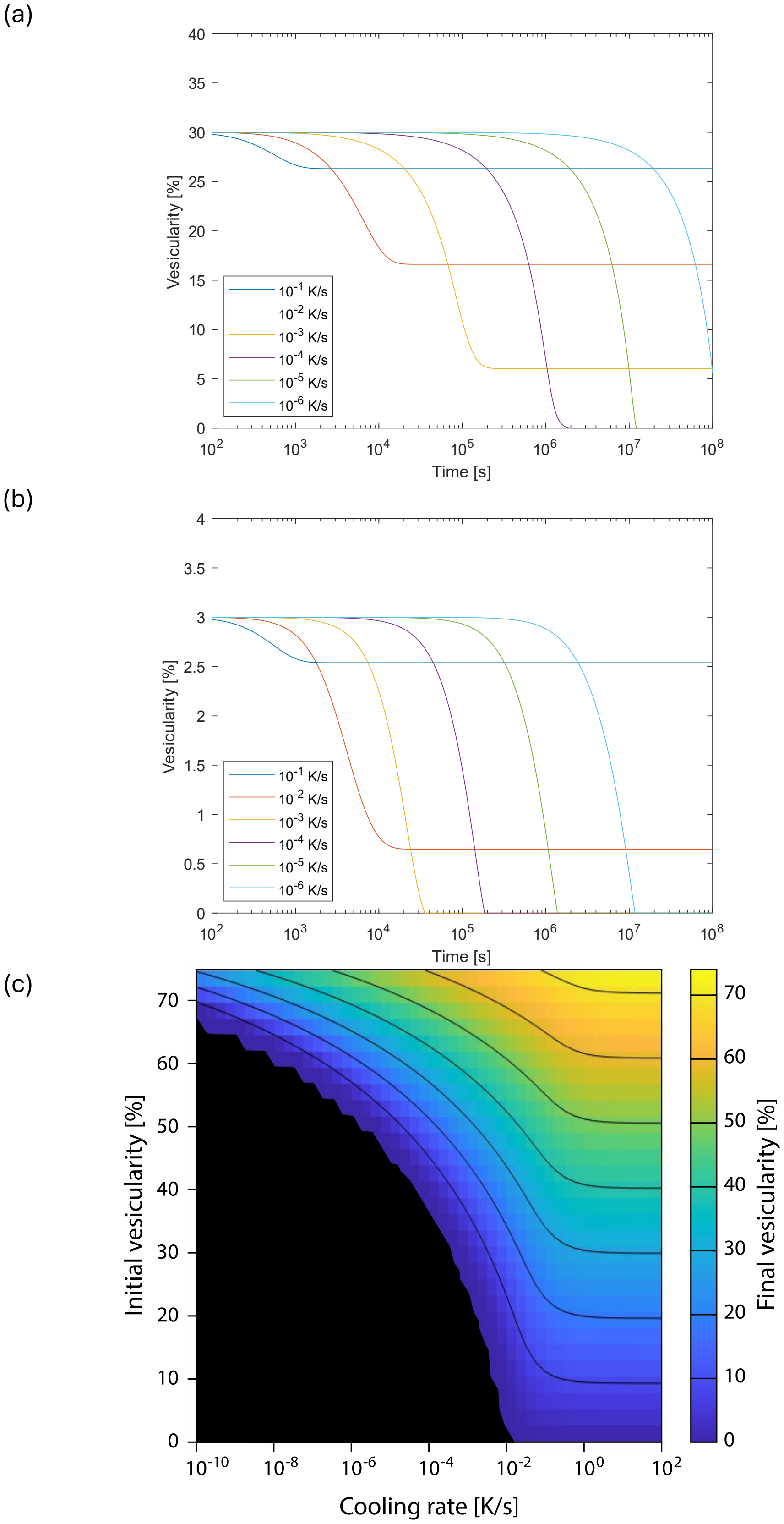

Supplementary Figure 3. Results of numerical modelling. *(a, b) Evolution of vesicularity over time for different cooling rates, for rhyolite samples starting from 30 and 3 vol. % vesicularity. (c) Map of initial and final vesicularity for rhyolite samples cooled at different rates. See Table S3 for conditions.*

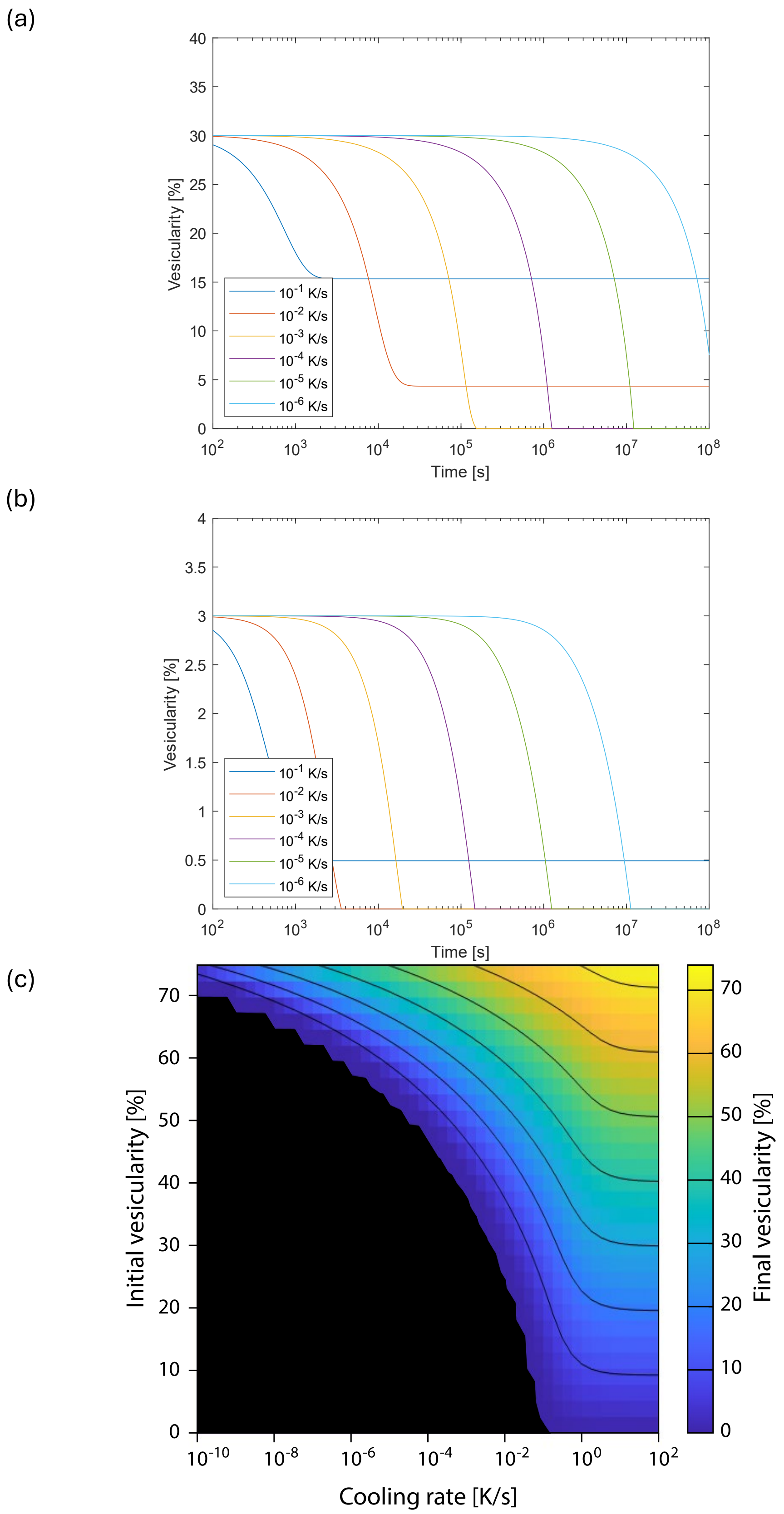

Supplementary Figure 4. Results of numerical modelling. *(a, b) Evolution of vesicularity over time for different cooling rates, for rhyolite samples starting from 30 and 3 vol. % vesicularity. (c) Map of initial and final vesicularity for rhyolite samples cooled at different rates. See Table S3 for conditions.*

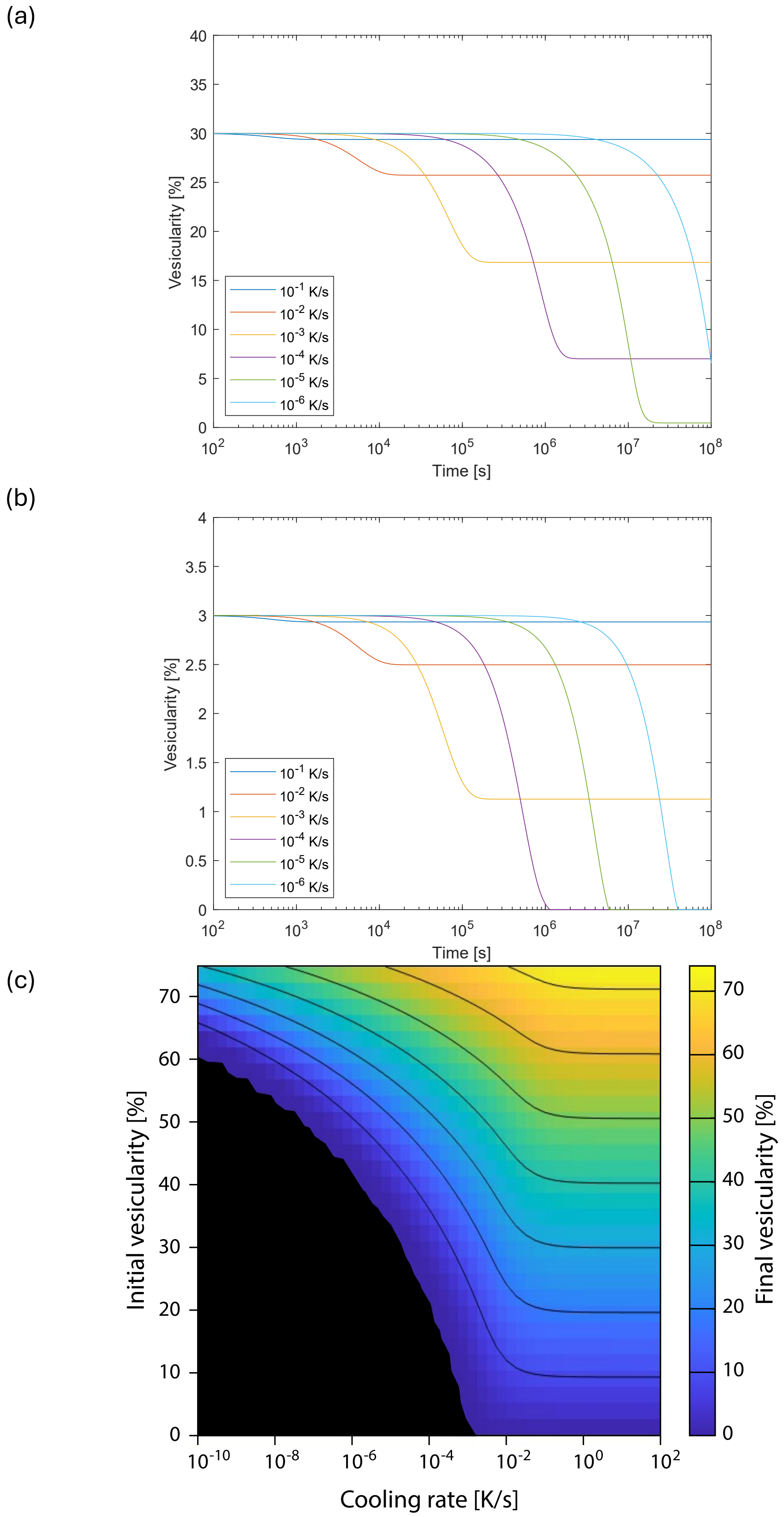

Supplementary Figure 5. Results of numerical modelling. *(a, b) Evolution of vesicularity over time for different cooling rates, for rhyolite samples starting from 30 and 3 vol. % vesicularity. (c) Map of initial and final vesicularity for rhyolite samples cooled at different rates. See Table S3 for conditions.*

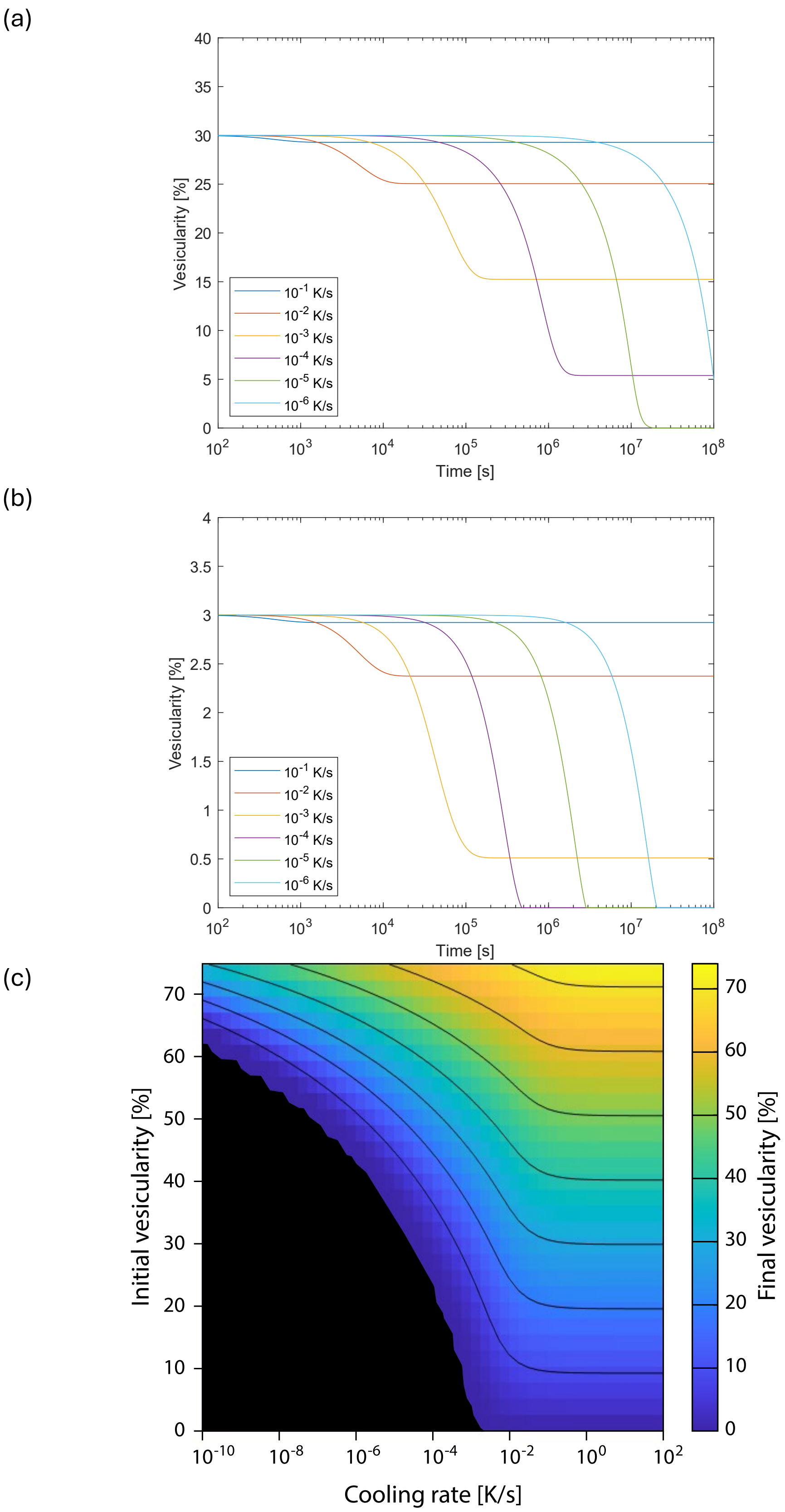

Supplementary Figure 6. Results of numerical modelling. *(a, b) Evolution of vesicularity over time for different cooling rates, for rhyolite samples starting from 30 and 3 vol. % vesicularity. (c) Map of initial and final vesicularity for rhyolite samples cooled at different rates. See Table S3 for conditions.*
